# Using Medical Device Standards for Design and Risk Management of Immersive Virtual Reality for At-Home Therapy and Remote Patient Monitoring

**DOI:** 10.2196/26942

**Published:** 2021-06-03

**Authors:** Joseph Peter Salisbury

**Affiliations:** 1 Playhab R&D Cognivive, Inc. Davis, CA United States

**Keywords:** virtual reality, telerehabilitation, remote patient monitoring, medical device design, safety, medical device regulation, risk assessment, failure modes and effects analysis

## Abstract

Numerous virtual reality (VR) systems have received regulatory clearance as therapeutic medical devices for in-clinic and at-home use. These systems enable remote patient monitoring of clinician-prescribed rehabilitation exercises, although most of these systems are nonimmersive. With the expanding availability of affordable and easy-to-use head-mounted display (HMD)-based VR, there is growing interest in immersive VR therapies. However, HMD-based VR presents unique risks. Following standards for medical device development, the objective of this paper is to demonstrate a risk management process for a generic immersive VR system for remote patient monitoring of at-home therapy. Regulations, standards, and guidance documents applicable to therapeutic VR design are reviewed to provide necessary background. Generic requirements for an immersive VR system for home use and remote patient monitoring are identified using predicate analysis and specified for both patients and clinicians using user stories. To analyze risk, failure modes and effects analysis, adapted for medical device risk management, is performed on the generic user stories and a set of risk control measures is proposed. Many therapeutic applications of VR would be regulated as a medical device if they were to be commercially marketed. Understanding relevant standards for design and risk management early in the development process can help expedite the availability of innovative VR therapies that are safe and effective.

## Introduction

### Virtual Reality as a Medical Device

Therapeutic virtual reality (VR) offers tremendous potential to provide innovative treatments in a broad range of clinical areas, including mental health disorders [1] (eg, traumatic stress [2,3], anxiety disorders [4], depression [5], schizophrenia [6], eating disorders [7]), pain management [8,9], motor and cognitive rehabilitation of neurodegenerative disorders [10,11], traumatic brain injury [12], stroke [13,14], and cognitive disorders [15,16].

While a wide variety of approaches have been referred to as VR in the literature, VR is popularly understood to include the use of a wearable head-mounted display (HMD) that creates a sense of being immersed in a virtual environment. HMD-based immersive VR has only recently begun to approach the same level of affordability as nonimmersive VR. The sense of presence that immersive VR offers has considerable potential to differentiate the impact of VR in clinical contexts, including telerehabilitation, moving forward [17-19]. Critically, immersive VR offers the potential for greater ecological validity in therapy, allowing the brain to respond to stimuli similar to how it does in the real world [20-24].

As VR interventions are developed and evaluated by clinicians and patients—particularly in an at-home environment—it is essential to evaluate the regulatory requirements that may restrict the translation of such technologies to routine clinical practice. For VR interventions that will be classified as a medical device, it is strongly recommended that requirements be identified early in the design and development phase to prevent costly reworkings of the system, software, and associated documentation [25-27].

While VR interventions include both a hardware and software component, many proposed VR interventions (particularly those for at-home use) leverage off-the-shelf (OTS) VR technology. In the most recent wave of technology, this includes the standalone VR headsets with 6-degrees of freedom (6-DOF) tracking, including the Vive Focus Plus, Oculus Quest, and Pico Neo. While designed primarily for nonmedical purposes, this does not restrict their use as a component of a medical device. A consumer VR headset is transformed into a medical device by virtue of the intended use of the software it is running. While additional, built-for-purpose hardware components may be introduced into a therapeutic VR system (eg, custom sensors, adaptive controllers), the software component is necessary and essential for transforming OTS VR devices into a medical device, and thus, can be considered as part of the larger category of software as a medical device (SaMD) [28].

While the design, development, testing, and postmarket surveillance of therapeutic VR include many of the same considerations, HMD-based VR presents unique challenges in comparison to the broader category of SaMD. In addition to the potentially hazardous situations introduced by wearing an occlusive headset that can induce side effects ranging from simulator sickness [29,30] to seizure, fully immersive VR introduces novel challenges for interface design among a population that will typically have little-to-no experience with the technology [31-33]. Thus, it is becoming useful to discuss the requirements of VR as a medical device (VRaMD) in their own right.

### Medical Device Quality Requirements

To understand what regulatory requirements may be for a given VR intervention, it is important to first consider whether the intended use is indeed classified as a “medical device” in a particular jurisdiction. The Global Harmonization Task Force published a guidance document toward an internationally recognized definition of a medical device [34]. In the United States, a medical device is defined in section 201(h) of the Food, Drug, and Cosmetic Act [35].

The US Food and Drug Administration (FDA) has published guidance [36] that outlines certain software functions that may meet the definition of a medical device, but as they pose a lower risk to the public, the FDA intends to exercise enforcement discretion. That is, the FDA will not enforce medical device regulatory requirements on this software. Included in the type of software is one that “use video and video games to motivate patients to do their physical therapy exercises at home.” With that said, this guidance document also states that software becomes a regulated medical device by performing patient-specific analysis and providing patient-specific diagnosis or treatment recommendations. Furthermore, there are specific regulatory classifications in the United States that classify “interactive rehabilitation exercise devices” as Class II medical devices, providing a clear regulatory path for a VRaMD intended to provide rehabilitation. Ultimately, manufacturers interested in commercialization in the United States are encouraged to contact the FDA to determine what, if any, regulatory requirements may apply.

Assuming the intended use of a VRaMD is determined to be a regulated medical device in a particular jurisdiction, it is important to understand regulatory requirements early in the device and development process. When developing a novel medical device, those without a background in medical device engineering may assume the burden to demonstrate the safety and effectiveness of a medical device is the domain of clinical investigators. However, it is important to note that the universal expectation of regulatory bodies is that safety and effectiveness be built into the system in early design and development stages. In the United States, Title 21 of the Code of Federal Regulations (CFR) [37] provides specific regulations that define the minimum current good manufacturing practice (cGMP) requirements for drugs, biologics, and medical devices. The cGMP regulations, also known as the quality system regulation (QSR), are based on the “quality-by-design” principle, which calls for quality to be built into the product, as testing alone cannot be relied on to ensure product quality [38].

cGMP regulations require establishment of a quality management system (QMS). The QMS impacts an organization’s daily activities at every level, including product planning, design, development, testing, and change management. Software professionals coming from a nonregulated software development industry may find it difficult to adapt to the planning and documentation requirements imposed by quality requirements [39,40]. Quality requirements for medical device software development may seem to conflict with agile software development methodologies and impose a large amount of overhead when developing medical device software [41]. Still, it is critical that software professionals confront the challenge of medical device quality requirements head on not only to be compliant with regulations, but also to ensure medical device software is safe and effective for its intended purpose. For medical device software, there are clear expectations for how to document the entire software development life cycle, from establishing user needs through to verification, validation, postmarket surveillance, and change management.

Quality requirements for medical devices include the integration of risk management across the product life cycle. As a component of risk management, a systematic risk assessment for a device must be performed with risk controls implemented and verified to mitigate unacceptable device hazards. Implementing risk management as part of the requirements analysis and design process of an SaMD can aid in improving designs early in the development process. This can prevent the need for reworking solutions and changing project scope late in the development process when changes can be more costly. In the case of home-use VRaMD, risk analysis can reveal new system requirements that can help improve system usability and adoption while mitigating risks to patients.

### Objectives of This Paper

This paper reviews regulations, standards, guidance documents, and technical reports that can be relevant for the design and development of a VRaMD. To demonstrate the application of these standards in the design and development process, the requirements for a generic home-use VRaMD system for at-home therapy are specified. A risk assessment is performed on the requirements to derive a set of risk control measures. Methods for verifying these risk control measures are discussed. The objectives of this paper are to:

1. Provide an overview of medical device standards that are applicable to the development of VRaMD intended for home use and remote patient monitoring.
2. Analyze the requirements of a generic home-use VRaMD and demonstrate how risk management can be used to identify and evaluate hazards, determine appropriate risk control measures, and limit potentially hazardous situations.

## Design Standards Applicable to VRaMD

### General Quality Management System Requirements

Medical device regulations are the legally defined requirements within a jurisdiction for how medical device manufacturers must operate. Requirements for a particular medical device can be determined by classifying the device within the risk-based classification system of a particular jurisdiction. One of the most fundamental requirements of a medical device organization is implementation of a QMS [42]. A QMS is a formal system that documents policies, procedures, and responsibility to manage product or process quality. QMS requirements are specified by regulatory bodies to ensure medical devices will be safe and perform as intended. It is important to note that while QMS regulations and standards outline a range of specific requirements, they are typically broadly defined to allow a variety of ways an organization can achieve their goals. Thus, the scope and complexity of an organization’s QMS can vary widely depending on the device type, organization size and structure, and the nature of specific regulatory requirements.

While the requirement that a QMS be certified varies depending on regulatory jurisdiction and device type, to achieve broad recognition many manufacturers follow ISO 13485:2016 [43]. ISO 13485 specifies requirements for a QMS that can be used by an organization involved in one or more stages of the life cycle of a medical device. These stages can include design, development, and production of a medical device, as well as storage, distribution, installation, technical support, servicing, decommission, and disposal. In the United States, adherence to ISO 13485 is not required, although the US QSR is generally aligned with this standard.

An important aspect of the QMS relevant for VRaMD development is the concept of design controls. Design controls are a set of policies and practices intended to ensure consistent translation of input requirements into a product that meets those requirements. Both ISO 13485 and FDA QSR set out a series of requirements for design controls. Design control is an iterative process following a structured methodology to ensure the device under development will be safe, effective, and meet end-user needs. The design control process is often illustrated with the V-model [44]. Design control requirements specify a general framework where various deliverables are generated and approved at each stage of the design and development process through to device verification and validation activities. These deliverables are necessary for auditing the QMS and meeting regulatory needs, requiring a robust system of procedures for maintaining documentation and approvals.

While the expectations of the QMS design controls are well-defined, there remains considerable room for how an organization decides to carry out these objectives. As part of QMS requirements, it is expected that an organization establishes detailed design and development plans for each product. These plans should specify how the development process is carried out, including assignment of responsibilities to adequately trained personnel and how these procedures are aligned with regulatory requirements and appropriate standards.

### Risk Management for Medical Devices

As part of fulfilling regulatory requirements, organizations must perform risk management activities. For example, under the 2017 European Union Medical Device Regulation (EU MDR) [45], manufacturers must have a documented risk management plan, identify and analyze the known and foreseeable hazards for each device, estimate and evaluate the associated risks, and eliminate or control those risks. Risk analysis is required as part of the US FDA’s design control requirements (21 CFR 820.30) [46] and is a component of FDA premarket submissions. ISO 14971:2019 [47], recognized worldwide by regulatory bodies, is widely acknowledged as the principal standard for this purpose. As part of ISO 14971, an organization develops a risk management plan, which includes how device risk assessments should be conducted.

ISO 14971 describes the requirements of a risk management process for medical device development, including 6 key stages: risk analysis, risk evaluation, risk control, evaluation of overall residual risk acceptability, risk management report, and production and postproduction information. Like quality management requirements, the details of how these processes are carried out in practice are left to the manufacturer. To implement ISO 14971, a company must first establish and document how they will conduct a risk management process that includes the required components in the standard. To accomplish risk analysis, Annex G of ISO 14971 provides guidance on some techniques, including preliminary hazard analysis, fault tree analysis, and failure modes and effects analysis (FMEA).

FMEA enables any effect or consequence of individual components to be systematically identified and is more appropriate as the design matures [48]. FMEA can be applied during the design process to understand the impact of potential defects and incorporate changes relatively early when they are less expensive to make. Thus, safety is improved and performance is enhanced by minimizing the probability and severity of hazardous situations.

It is important to note that, although FMEA is a recognized risk assessment tool specified in ISO 14971, completing FMEA according to the FMEA standard IEC 60812:2018 [49] does not fulfill all the requirements of ISO 14971. For example, FMEA focuses on defects, whereas the focus of ISO 14971 is on harm. In ISO 14971, both normal and abnormal circumstances must be considered, as opposed to a focus on failure situations in FMEA. That is, even when the device is functioning as intended, hazardous situations may still occur, which must be identified. For example, the device may function as intended, but a specific subset of patients may experience side effects. Patients may also misinterpret instructions or feedback provided by the system. Of course, hazardous situations that arise from system malfunction, such as damage or misuse of the system leading to degraded system performance, must also be considered.

**Figure 1:**
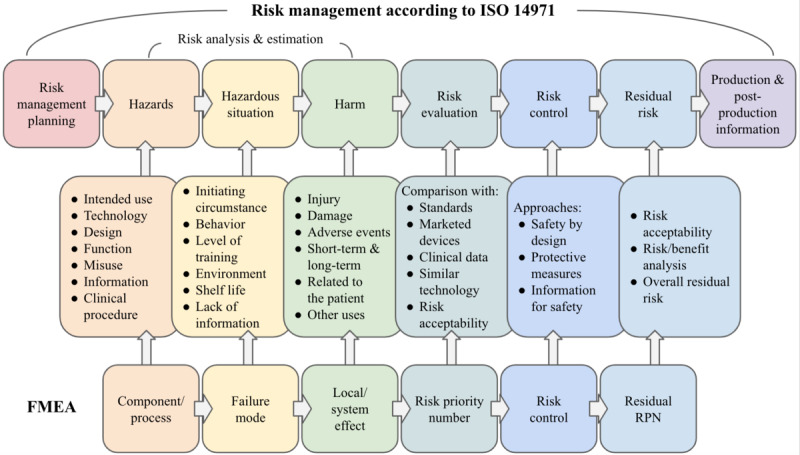
Overview of ISO 14971 risk management process requirements and how FMEA can be adapted. Redrawn and adapted from resources developed by Gantus and Semoegy (unpublished data). FMEA: failure mode and effects analysis; ISO: International Organization for Standardization; RPN: risk priority number.

Furthermore, FMEA can allow for low-priority defects to persist, whereas risks in a medical device should be reduced or eliminated as far as reasonably possible before a medical device can be marketed. Both ISO 14971 and FMEA require the risk parameters of occurrence and severity to be addressed, where occurrence is the probability of occurrence of harm and severity is the extent of its impact or consequences. However, FMEA also considers the probability of detecting the harm before it occurs, which is not part of ISO 14971. Harm may still happen even if it is detected, and harms not easily detectable may unnecessarily raise risk levels. Thus, this parameter is excluded. Once the differences between FMEA and ISO 14971 are understood, it is possible to adapt FMEA to meet the requirements of ISO 14971 (Figure 1).

To conduct the risk management process, the first step is to identify the hazards, hazardous situations, and associated harms of a device. Hazard identification can be performed by reviewing the medical device characteristics, such as intended use, technologies used in the device, how the device is intended to function in clinical procedures, what could occur if the device is misused, and what could occur if information from the device is misinterpreted.

Once hazards are identified, for each hazardous situation, risk estimation is performed whereby the probability of occurrence and severity of that harm is estimated. It is the responsibility of the manufacturer to establish an appropriate quantitative or qualitative method for categorizing probability of occurrence of harm and severity of harm. Tables 1 and 2 provide example ways of categorizing severity of harm and probability of harm occurrence. Note that these tables are intentionally kept simple for illustration purposes and could include greater (or fewer) categories, as appropriate.

**Table 1:** Example severity table.

Rank	Description	Criteria
5	Critical	Loss of limb or life-threatening injury.
4	Major	Severe, long-term injury; potential disability.
3	Serious	Short-term injury or impairment requiring additional medical intervention to correct.
2	Minor	Slight inconvenience with little to no effect on product performance; minor injury not requiring medical intervention.
1	Negligible	No significant risk of injury to patient.

**Table 2:** Example probability of occurrence table.

Rank	Description	Criteria
5	Frequent	1 in 100
4	Probable	1 in 1000
3	Occasional	1 in 10,000
2	Remote	1 in 100,000
1	Improbable	1 in 1,000,000

Acceptable methods for estimating risks are provided in ISO 14971 and include published standards, scientific technical data, field data from similar devices, usability tests, clinical evidence, and expert opinion. It is often not practical to assign numerical estimations for the likelihood of an occurrence of a particular harm. Thus, following qualitative descriptors can provide a reasonable method for estimating the probability of occurrence in the absence of precise data.

**Figure 2:**
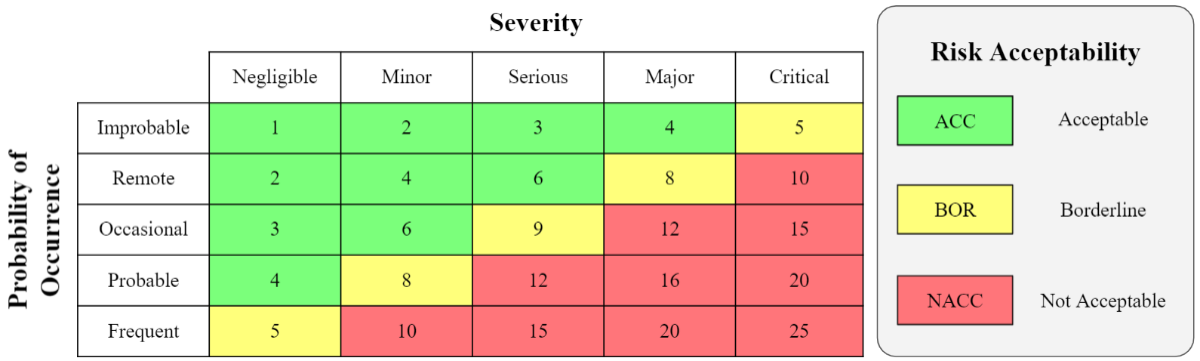
Risk evaluation matrix with risk priority numbers (RPNs) generated when multiplying the severity of harm rank (Table 1) with the corresponding probability of occurrence rank (Table 2). The risk evaluation matrix is divided into 3 risk regions, with acceptable risks in green, unacceptable risks in red, and borderline risks in yellow.

The “Rank” column is included in Tables 1 and 2 so that, following the FMEA approach, a risk priority number (RPN) can be generated for risk evaluation. In Figure 2, a risk evaluation matrix is generated by multiplying the probability of occurrence ranks with the severity of harm ranks. The risk evaluation matrix is divided into 3 risk regions to define acceptable risks (green), borderline risks (yellow), and unacceptable risks (red). Again, the illustrated risk regions are provided merely as an example, and a manufacturer can establish their own way of delineating acceptable and unacceptable risks, as may be appropriate for their device.

Hazards that are evaluated to have an unacceptable risk level require risk control measures. Borderline risks may also require risk control measures upon further investigation. While not required, risk control measures may also be desirable for acceptable risks as these may still improve the safety and performance of the device and lead to better end-user satisfaction. RPNs provide a way to prioritize the allocation of limited resources within a particular risk region.

Risk control measures defined in ISO 14971 include inherent safety by design, protective measures in the medical device itself or in the manufacturing process, and information for safety. Implementation and effectiveness of risk control measures must be verified and validated by the manufacturer. To evaluate the effectiveness of risk control measures, it is often necessary to conduct usability tests. For example, if information for safety is utilized, it is important that information is perceivable, understandable, and supports correct use of the device by the intended user group in the context of its intended use environment. IEC 62366-1 [50] is an international standard that can be used with ISO 14971 to conduct these evaluations. The US FDA has also developed their own guidance document [51].

After risk control measures are applied, any residual risk is required to be evaluated. Residual risk that is judged not to be acceptable requires further risk control measures. In the event residual risk is not acceptable and further risk control is not practicable, the manufacturer may conduct a risk–benefit analysis by gathering and reviewing data and literature to determine if the medical benefits of the intended use outweigh the residual risk. Information for safety may be used by the manufacturer to disclose risks that may outweigh the benefits of the device.

### Software Life Cycle Processes

The majority of software problems are traceable to design and development errors, making software design control critical [52]. In both the United States and EU, all software components must be under design control or purchasing control, including design validation that includes software validation and risk analysis [53]. The EU adopted a new essential requirement regarding software in 2007, Essential Requirement 12.1.a [54], addressing the software life cycle. In EU MDR, safety and performance requirements (SPRs) replace the essential requirements and SPR 17 places greater emphasis on the entire product life cycle, as well as introducing specific requirements for mobile computing platforms and information security. Likewise, the US FDA has guidance on general validation principles applicable to medical device software [55].

IEC 62304 [56] provides specific guidance on the processes to be performed for the development of medical device software, including risk management activities. IEC 62304 is an EU harmonized standard and is recognized by the FDA as an approved consensus standard and thus can be used as a benchmark to comply with both markets’ regulatory requirements. This standard provides a life cycle process framework, with activities and tasks necessary for safe medical development. A central theme of IEC 62304 is the need to establish and maintain traceability between system requirements, software requirements, software testing, and risk control measures implemented in software [57]. Established user needs provide the foundation from which all software requirements are derived and must be maintained throughout the product life cycle. It is important to note that while the V-model is often used to illustrate these requirements, a “waterfall” approach is not necessary. IEC 62304 clarifies incremental strategies (eg, Agile), which acknowledge user needs may not be fully defined or may evolve throughout the product life cycle, can still meet the requirements specified in the standard.

Ultimately, it is the responsibility of the manufacturer to define how user needs and system requirements are captured, refined, and tested during product development. Numerous resources are available to help with this process and provide a way for manufacturers to demonstrate traceability and generate reports necessary for regulatory compliance while benefiting from the value of Agile practices. For example, the Association for the Advancement of Medical Instrumentation (AAMI) has published a technical information report (TIR)—AAMI TIR45:2012 [58]—to help provide guidance on how best to align Agile and medical device regulatory perspectives to develop safe and effective medical device software. AAMI TIR45 covers key topics such as documentation, evolutionary design and architecture, traceability, verification and validation, management changes, and “done” criteria. While adopting the practices recommended in AAMI TIR45 is not strictly required, and there are certainly other sensible ways to adapt Agile methodologies for medical device development, the US FDA recognizes AAMI TIR45 as a consensus standard. This recognition provides assurance that Agile practices can be successfully adapted to meet regulatory compliance requirements.

An important feature of AAMI TIR45 is that it provides a framework for reconciling the user story approach [59] for incrementally specifying product requirements with the design input/design output framework used in medical device development. A user story is a short, simple description of a feature told from the perspective of the person who desires the new capability (ie, an end user of the system or other stakeholder). User stories are typically written following the template: *As a <type of user>, I want <some goal> so that <some reason>*. To elaborate on a story, an accompanying set of acceptance criteria can be specified. User stories can also be broken down into more specific user stories when necessary and appropriate. As AAMI TIR45 explains, the goal of a user story is to be persistent and lightweight, capturing just enough essence of a requirement to allow for future discussions to uncover or elaborate more when needed.

### Summary of VRaMD Design and Development Considerations

To summarize:

1. Depending on the regulatory jurisdiction and classification, VRaMD design and development may need to be captured in a QMS following design control requirements. This may include use of ISO 13485 or local regulations (eg, US FDA QSR).
2. For software development specifically, IEC 62304 provides a standard framework. AAMI TIR45 can used as guidance for adopting Agile practices, if desired.
3. Risk management according to ISO 14971 should be performed during the design process. FMEA is one risk analysis tool that can be adapted to ISO 14971. Effectiveness of risk control measures can be evaluated in usability tests following IEC 62366.

To demonstrate these concepts, a VRaMD for home use and remote patient monitoring is specified using a set of generic user stories. Applying FMEA, hazards associated with each user story are identified and risk is evaluated based on estimations of probability of occurrence and severity of harm. Risk control measures are proposed, and the residual risk is determined to demonstrate how a safe and effective VRaMD may be designed.

## Risk Management for a Generic VRaMD

### Requirements Analysis

To specify the requirements for an at-home VR rehabilitation system, it is helpful to review similar devices that are already legally marketed. In the United States, this is a common regulatory strategy. Demonstrating substantial equivalence to a legally marketed device, referred to as a predicate device, enables many devices to be cleared under the premarket notification [510(k)] submission process. In the US FDA medical device classification scheme, devices are classified as Class I, II, or III based on risk level, with Class I devices presenting the lowest risk, and Class III devices presenting the highest risk. Within each FDA class, device types are classified within regulations, which include special control requirements for Class II devices. The intended use and technological characteristics of a system obtaining 510(k) clearance are often made publicly available as a “510(k) Summary” (21 CFR 807.92).

Over the past decade, numerous devices that included at-home physical rehabilitation using video game technology received 510(k) clearance. This began with Jintronix (Jintronix Inc.) [60-62] and went on to include the Recovr Rehabilitation System (Recovr, Inc.) [63], Vera (Reflexion Health, Inc.) [64], the Yugo System (BioGaming Ltd.), the Virtual Occupational Therapy Application (Barron Associates, Inc., marketed as SaeboVR by Saebo, Inc.) [65-67], Uincare Home (UINCARE Corp.) [68], and MindMotion Go (MindMaze SA) [69]. These devices are regulated as Class II devices in the United States. None of these systems utilize HMD-based VR, but rather the Microsoft Kinect motion tracking system. Still, each system includes the intended use of supporting physical rehabilitation of adults at-home by providing therapy guidance for patients and remotely accessible performance metrics for medical professionals.

**Figure 3:**
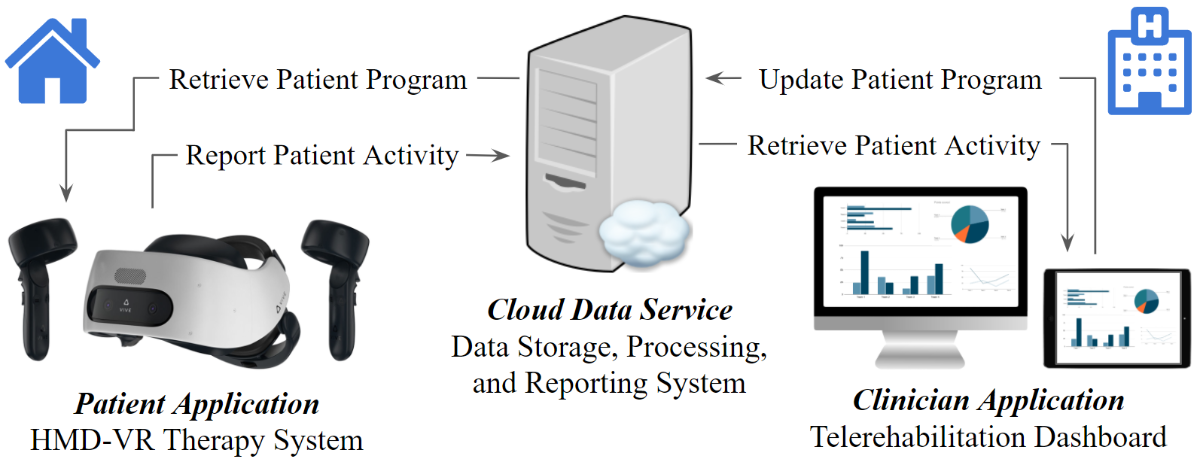
Overview of generic VR as a medical device system for home-use and telerehabilitation. HMD: head-mounted display; VR: virtual reality.

By examining 510(k) Summaries for these devices, it can be seen they generally include 3 separate applications: a patient-facing application, a clinician-facing application, and a cloud-based server for providing data storage and managing communication between the 2 applications. The patient-facing application (henceforth, patient application) prompts and monitors patients in the performance of a therapy prescribed by their clinicians, reports performance data to the clinician for analysis, and provides an interface for patients to communicate with their clinician. The clinician facing-application (henceforth, clinician application) allows a clinician to define and update a patient’s personal data, a patient’s therapy prescription, monitor a patient’s performance of that therapy, and permit a clinician to communicate with a patient. Thus, the common components of a VR telerehabilitation system have been established. Taking these descriptions into account, the core functionality for an immersive VR therapy system can be specified by replacing the Kinect with a standalone HMD-based VR system with 6-DOF tracking (Figure 3). A generic set of user stories for the patient application can be constructed from the descriptions of these systems (Table 3). Likewise, a generic set of user stories for the clinician application can also be constructed (Table 4). These generic user stories have provided a basis for designing additional therapy systems, including one using HMD-based VR [70,71].

**Table 3:** Overview of virtual reality system requirements—patient stories.

ID	Summary	As a patient I want...	so that...
P1	Trusted exercise	to trust that exercises I perform have clinical utility	I know the time I spend using the system is worthwhile and will benefit my health.
P2	Ease of use	to feel confident I will be able to use the system at-home with minimal assistance	I can effectively benefit from the system and recover independently.
P3	Portability	to be able to perform therapy at home	I do not have to rely on transportation and scheduling and can recover on my own.
P4	Clinician supervision	my clinical health care provider to have access to my therapy data	communication regarding my at-home therapy is streamlined.
P5	Instructions	to have clear instructions on how to perform exercises	I am reminded how to properly complete these actions.
P6	Feedback	to have feedback as I am performing exercises	I know if I am performing exercises effectively.
P7	Motivation	exercises to feel more like a video game than homework	I am motivated to adhere to prescribed exercises.
P8	Performance summary	to see the results of my exercise performance	I know if I am performing exercises effectively based on established targets.
P9	Track progress	see my progress over time	I am intrinsically motivated to advance my recovery.
P10	Reminders	to be reminded to do exercises	I do not forget and take longer to recover.

**Table 4:** A generic set of clinician requirements for a virtual reality as a medical device system designed for home use and remote patient monitoring.

ID	Summary	As a clinician I want...	so that...
C1	Clinical validity	to know how system routines correspond to clinically valid therapies	I know how to apply my current understanding of therapy to the system’s functionality.
C2	Instructions	to be able to learn the system quickly and completely	I am confident I understand how to utilize the system and integrate it into my practice without too much difficulty.
C3	Ease of use	to be able to quickly train patients and caregivers on the system	I feel confident they will be able to utilize the system at home.
C4	Manage patients	an online dashboard to manage my patients	I can add patients to the system.
C5	Personalization	to be able to give each patient their own profile	I can customize individual patients and track their progress over time.
C6	Customization	to be able to specify exercises for patients	I can target areas where patients need improvement.
C7	Assessment	to be able to assess patient ability	I can determine a baseline challenge level that can be used to monitor progress.
C8	Reminders	to remind patients to complete therapy	patients can be re-engaged if they are not active.
C9	Adherence tracking	to know when patients complete therapy	I know if patients are adhering to recommended frequency.
C10	Exercise tracking	to know how much patients complete therapy	I know if patients how much patients accomplish and whether they are being adequately challenged.
C11	Movement tracking	to know how patients perform treatment	I know how patient’s exercise and if they are using clinically valid movements.
C12	Progress tracking	to know how well patients perform exercises	I know a patient’s ability at a given time.
C13	Symptom tracking	to know how patient symptoms change (eg, improve)	I know if the patient can progress in a therapy.
C14	Remote feedback	a way to provide feedback to patients	I can communicate with patients, if necessary.
C15	Exportability	a way to export patient performance records (eg, through a printable report)	I can share clinically interpretable data with other members of the patient’s clinical care team and with payers.
C16	Cybersecurity	my patients’ records to be secure and protected	I can manage protected health information responsibly.

### Risk Analysis and Evaluation

For each user story, the adapted FMEA process can be used to identify hazards (ie, potential sources of harm) of that feature (Multimedia Appendix 1). Hazards can lead to hazardous situations which may then cause harm. As part of the risk analysis, the probability of occurrence of harm is estimated based on a combination of the likelihood of both the hazard and the hazardous situation. Then, the severity of the harm produced by each hazardous situation is considered. For example, the display screens used in HMD-based VR can be considered a consistent hazard, which may lead to a variety of different hazardous situations. When identifying hazardous situations, it is important to consider both normal and abnormal use of the device. Likewise, hazards can still cause harm without a device failure. Even when using HMD-based VR as intended, there is potential for side effects including eye strain, claustrophobia, overstimulation, anxiety, and seizures. On one extreme, eye strain may be considered a common side effect of using HMD-based VR. However, the severity of resulting short-lasting headaches may be considered low. Alternatively, patients with photosensitivity may experience seizures. While the probability of this occurrence is much lower, if the patient is using the system independently at home, this seizure could fatal, and thus, critically severe.

**Figure 4:**
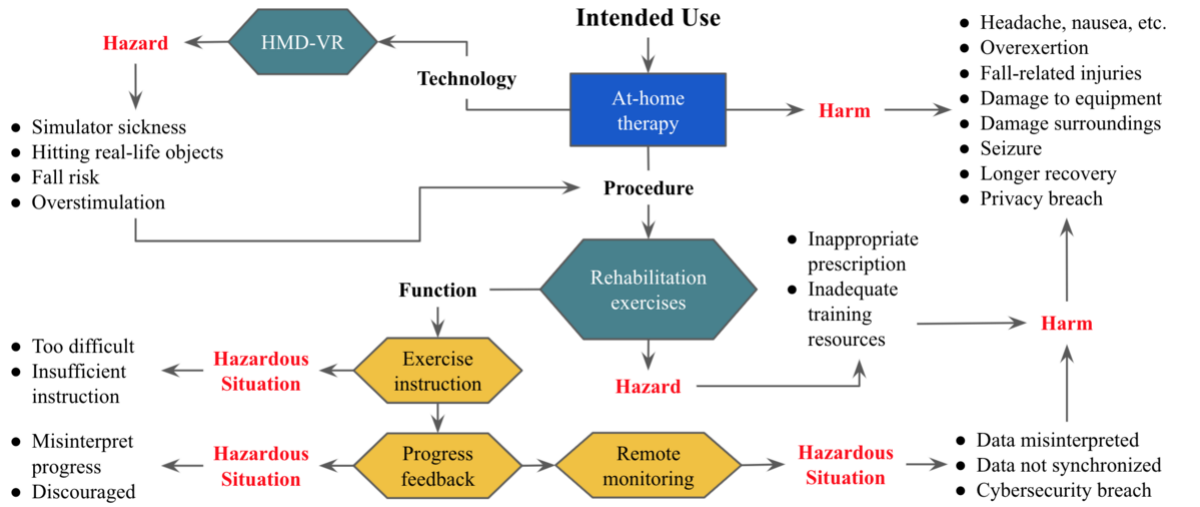
Risk analysis summary for a head-mounted display-based virtual reality (HMD-VR) medical device for home use and telerehabilitation.

In addition to the hazardous situations related to HMD-VR specifically, Multimedia Appendix 1 lists many hazards that would be common to non-HMD-VR at-home therapies. For example, data failing to properly synchronize between the clinician and patient applications can lead to the patient not receiving proper treatment. Hazardous situations also arise whenever either user group is unable to properly interpret instructions or data provided to them. This is most severe when it leads to the patient not being able to benefit from treatment. Thus, it can be seen how critical usability engineering can be to ensure proper medical device function. Figure 4 summarizes the identified risks associated with the system.

### Risk Control Measures and Residual Risk Evaluation

ISO 14971 defines “safety” as freedom from unacceptable risk. Based on the risk assessment and acceptability criteria defined earlier, several unacceptable risks were identified that require risk control measures. A variety of borderline risks were also identified, which would require further investigation to determine whether risk control measures are necessary. While the remaining hazards were deemed to have an acceptable risk level, these potential failure points highlight opportunities to improve the system design and create greater patient and clinician satisfaction with the system. Thus, in Multimedia Appendix 2, risk control measures have been proposed for all hazards.

**Figure 5:**
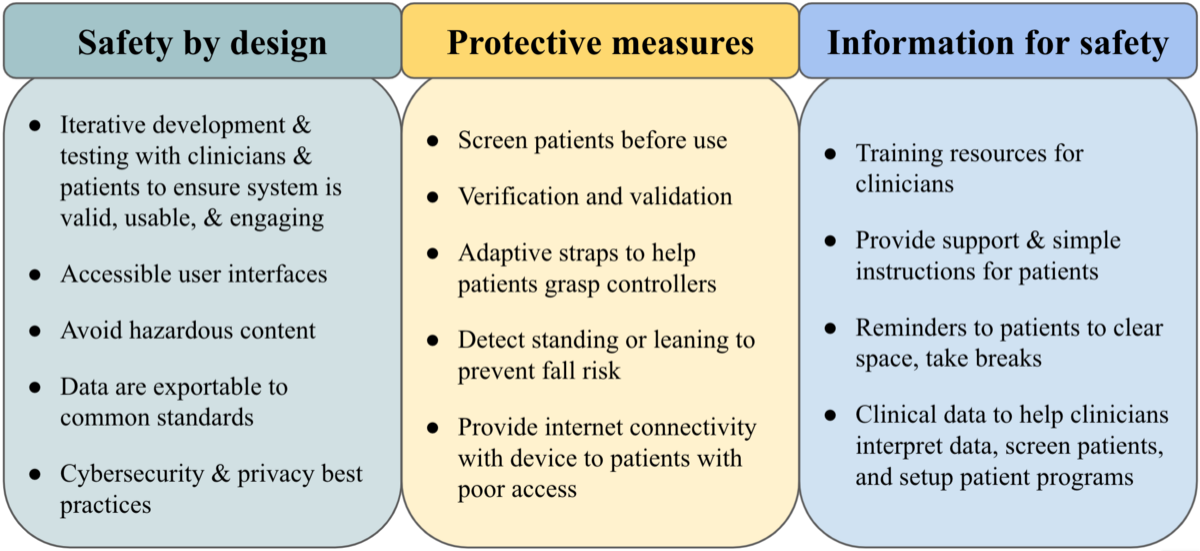
Summary of risk control measures to improve safety and end-user satisfaction.

After completing the residual risk evaluation, almost all risks have been brought within the acceptable region. The only remaining borderline risk is associated with the potential for seizures in patients with photosensitivity. While the likelihood of a patient with these issues can be reduced by prescreening patients for a history of seizures, the harm associated with this situation is still considered severe. Overall, an HMD-based VRaMD can be designed to be safe for at-home use and remote patient monitoring when risk control measures are applied. Figure 5 summarizes the risk control measures that are recommended.

When examining the potential sources of unacceptable risks, important areas to consider early in the design and development of a VRaMD can be identified. For example, one source of unacceptable risk is related to insufficient clinician training resources, which can lead to a clinician not understanding the proper intended use of the system and prescribing it to inappropriate patients; clinicians not understanding how to properly configure the system to meet patient needs; and clinicians not understanding how to train patients on the system. To control for this risk, usability testing with clinicians should verify the system’s intended use is understandable and meets clinician needs. For clinicians to be able to utilize the system, they must both understand how to develop individualized patient treatment plans and interpret patient data generated by the system. Furthermore, there should be adequate resources available for clinicians to be able to train patients on the system, if necessary. Likewise, usability testing with patients should verify patient application exercise instructions are adequate to elicit target therapeutic actions. Resources specific to VRaMD usability evaluations should be considered [72]. System feedback provided to patients should also be interpretable by patients and, ideally, provide motivation so patients do not become discouraged by their results for whatever reason.

Hazardous situations can also occur when data between the patient and clinician applications can not properly synchronize due to internet connectivity issues. Depending on the severity of harm, it may be necessary for an internet connection to be provided as part of the system to ensure data can synchronize. This could also alleviate risk caused by patients not being able to connect the device to their home network. However, if a stable internet connection is available, it may be sufficient to provide adequate instruction and have a remote provider verify the patient has completed the necessary set up.

Finally, cybersecurity and patient privacy issues must be addressed. Indeed, protections for these concerns are critical for adoption of a connected health technology [73,74].

### Cybersecurity and Patient Privacy Protections

Regulatory bodies generally require cybersecurity considerations as an aspect of the device design and risk management process. MDCG 2019-16 [75] provides guidance on how to fulfill essential requirements regarding cybersecurity specified in Annex I of EU MDR. Likewise, the US FDA provides guidance regarding cybersecurity information to be included in FDA premarket submissions [76], as well as guidance for postmarket management of cybersecurity vulnerabilities [77]. While it is generally up to the manufacturer to determine what cybersecurity controls are necessary for their device, applying recognized standards can help demonstrate implemented capabilities are appropriate and effective.

A variety of standards for addressing medical device cybersecurity are available to help manufacturers ensure they are following industry best practices. Selecting which standards to adopt can depend on the specific technologies and interfaces used by a product (see [78] for further discussion). One starting point is ANSI/AAMI/IEC TIR80001-2-2:2012 [79], which presents an informative set of high-level security capabilities that are intended to facilitate more effective communication of security requirements with stakeholders. The Manufacturer Disclosure Statement for Medical Device Security (MDS2) form [80] is aligned with the security capabilities described in TIR80001-2-2 and provides manufacturers a format for reporting the data assets handled by a medical device, as well as the approach taken to secure it. The MDS2 form thus provides a way for manufacturers to disclose to health care organizations (eg, hospitals) information necessary for them to conduct their own cybersecurity risk analyses [81].

Once necessary security capabilities are identified through risk management and understanding stakeholder needs, AAMI/IEC TIR80001-2-8:2016 can be used to determine specific design requirements from a set of common security standards. This allows a design team to select appropriate standards, as well as provide evidence that each of the applicable security capabilities have been met [78].

When evaluating necessary cybersecurity capabilities (eg, data access controls) and associated procedures (eg, notification of patient’s data rights, notifications of detected data breaches to appropriate stakeholders), medical device manufacturers must understand and comply with the local legal requirements (eg, General Data Protection Regulation [GDPR], Health Insurance Portability and Accountability Act [HIPAA]). Regarding data rights and governance, manufacturers may employ end user license agreements, terms of service, and privacy policies to establish and convey company and user data rights for monitoring, evaluation, and distribution of collected data [73]. Additional precautions may be necessary for certain patient populations, such as children (eg, the Children’s Online Privacy Protection Act).

## Discussion

### Summary of Recommendations

HMD-based VRaMDs, depending upon their intended use, will likely be subject to the same regulatory requirements as other medical devices. Quality requirements such as design controls may be unfamiliar to product designers and software professionals coming from unregulated fields. While design control requirements may appear to suggest a “waterfall” approach is necessary, it is not incompatible with Agile practices, which can be used once properly adapted. Incorporating regulatory requirements early in the design process is not only necessary but also helps eliminate costly reworkings later in development. Incorporating a risk management process will help systematically expose ways to make the product safer and improve end-user satisfaction. A comprehensive usability engineering plan is necessary to verify risk control measures are effective.

Using non-HMD-based VR systems already legally marketed in the United States for at-home therapy, a generic set of user stories for both patients and clinicians was specified here. While HMD-based VR introduces unique hazards to at-home therapy, the associated risks can be mitigated with appropriate control measures, demonstrating that HMD-based VR can be designed to be safe for home use and remote patient monitoring.

For clinicians, it is important they understand the proper intended use of the system. This will enable them to prescribe the system to appropriate patients, understand how to configure the system to meet a particular patient’s needs, and be able to interpret system performance metrics as intended to progress a patient through treatment. This can be accomplished through robust user interface design and providing clinicians with the necessary training resources. The effectiveness of these measures should be verified in usability testing. Finally, the accuracy of OTS movement tracking sensors should be verified to be within clinically relevant ranges if 3D motion data are used in assessing patient performance. Studies have already evaluated the accuracy of the Oculus Touch controllers [82] and HTC VIVE motion tracking sensors [83] for clinical use in motor rehabilitation.

Patients must understand risks associated with HMD-VR so that they may avoid hazardous situations at home. General risks may be avoided by properly clearing the environment of obstacles, avoiding standing with the headset on, taking breaks to rest, and stopping use of the device if they experience negative effects. When instructing patients to perform therapeutic actions, it is important they have the necessary guidance and feedback to determine if they are performing the therapy as intended. When providing patients with progress data, it is important these data are easily interpretable. Ideally, performance feedback data should not discourage the patient from continuing treatment. To determine if system safety information is effective, usability testing in patients’ homes is necessary. An iterative human-centered design approach with clinicians and patients can help guide design concepts toward success early in development [84,85]. Assuming transmission of data between clinicians and patients is necessary for effective treatment, measures should be taken to verify the device is connected to the internet upon arrival at the patient’s home.

Finally, appropriate patient privacy and cybersecurity protections are essential. Standards can be utilized to determine necessary security measures and how to implement them effectively. Stakeholder needs, including relevant data privacy regulations, will contribute to the assessment of necessary cybersecurity capabilities. The MDS2 form provides one method for communicating with health care providers data handled by the system and how they are protected. End users should be provided with a privacy notice that describes how data are collected, used, and retained, the types of data that the product obtains, the length of data retention, and how and by whom information is used.

### Limitations

This paper describes a generic VRaMD system, using devices with a similar intended use as a basis. System functionality was specified at only the highest level to provide a reasonable scope for examination and discussion in the paper. Given a more specific intended use, more detailed requirements will be specified that may introduce new hazards to the system. The probability of occurrence and severity of device harms were roughly estimated for practical purposes.

A standalone HMD-based VR system with 6-DOF tracking was used as the core technology. Use of other VR systems may introduce different hazards. For example, a non-standalone system with external sensors (eg, Oculus Rift) may require additional set up and monitoring of sensor placement. More expensive head-mounted augmented and mixed reality systems (eg, Microsoft HoloLens, Magic Leap) were also not considered, although augmented and mixed reality–based medical devices are in development and may resolve certain hazards and limitations associated with occlusive VRaMD [86,87].

Overall, the intention of this paper was to provide an overview of an ISO 14971-compliant risk management process. To accomplish this, it was necessary to review related medical device regulations, standards, and guidance documents. While these requirements and recommendations are applicable to a variety of SaMD, specific devices and regulatory jurisdictions may require additional considerations. This paper was also not intended to be an exhaustive review of applicable standards. For example, the IEC 60601-1 [88] series of standards for electrical medical devices was not discussed. This was done to keep the focus of the paper on software and implementation of IEC 62304. However, IEC 60601-1 may be necessary for demonstrating the safety and electromagnetic compatibility of system hardware. More general (ie, nonmedical device specific) standards may also be useful for the design process, such as ISO 9241-210:2019 [89]. The IEEE Virtual Reality and Augmented Reality Working Group is developing standards for VR design that could be useful to apply to improve the safety, usability, and standardization of VRaMD [90]. Ultimately, a VRaMD manufacturer should communicate with the appropriate regulatory bodies when developing a new product intended for commercialization. The review provided here is intended to help orient those new to medical device development and provide a broad overview of regulatory requirements applicable to a variety of jurisdictions.

### Comparison With Prior Work

Recent advances in the widespread availability of VR and its potential in therapy have led to growing interest in the development of industry best practices for translating this potential to a reality. For example, the Virtual Reality Clinical Outcomes Research Experts (VR-CORE) committee has published a framework for iterative clinical trial design for validating VR therapies [91]. Numerous papers have also shared the design process for various HMD-based VR interventions [92-99]. The focus of this paper was less about the design of a particular system, and more about demonstrating a risk management process to develop a VRaMD safe for home use and remote patient monitoring.

Numerous reports have examined challenges and best practices for introducing medical device regulatory requirements, such as design controls and risk management, into contemporary software development practices such as Agile [39,40,100-110]. Here, Agile techniques were introduced primarily as a method for specifying the requirements of the system through user stories. Additional concepts were introduced as necessary to demonstrate the risk management process. It is expected that VR developers working on VRaMD will be coming from nonregulated fields (eg, video games, entertainment), and thus it is important to provide some necessary background. While the transition to medical device development can be challenging, this paper describes how Agile practices can be utilized to develop a safe and effective VRaMD. More recently, the HMD-based REAL Immersive system obtained 510(k) clearance for in-clinic use, providing further evidence that immersive VRaMD can successfully meet regulatory requirements.

### Conclusions

HMD-based VR offers tremendous potential for novel at-home treatments. However, for these treatments to be successfully translated into clinical practice, VRaMD will need to be designed following the necessary regulatory requirements. While regulatory requirements can appear challenging, VRaMD designers should find it beneficial to gain an understanding of what is required so they may adapt their design process early in development. While medical device design controls present a need for comprehensive documentation of device design, incorporating risk management early in this process should help further refine system requirements. Following these recommendations will help make VRaMDs safe and effective, as well as improve patient and clinician satisfaction with these novel digital therapeutics.

## Appendix

Multimedia Appendix 1 Risk analysis and risk evaluation of patient and clinician user stories.

Multimedia Appendix 2 Failure modes and effects analysis including risk control measures and residual risk analysis.

## Abbreviations

AAMI: Association for the Advancement of Medical Instrumentation
CFR: Code of Federal Regulations
cGMP: current good manufacturing practice
DOF: degrees of freedom
EU: European Union
FDA: Food and Drug Administration
FMEA: failure modes and effects analysis
HMD: head-mounted display
IEC: International Electrotechnical Commission
ISO: International Organization for Standardization
MDCG: Medical Device Coordination Group
MDR: Medical Device Regulation
MDS2: Manufacturer Disclosure Statement for Medical Device Security
OTS: off-the-shelf
QMS: quality management system
QSR: quality system regulation
SaMD: software as a medical device
SPR: safety and performance requirements
TIR: technical information report
VR: virtual reality
VRaMD: virtual reality as a medical device
